# Frequency of complementary and alternative medicine utilization in hypertensive patients attending an urban tertiary care centre in Nigeria

**DOI:** 10.1186/1472-6882-7-30

**Published:** 2007-09-28

**Authors:** Oluwatoyin C Amira, Njideka U Okubadejo

**Affiliations:** 1 Department of Medicine, College of Medicine, University of Lagos, PMB 12003, Lagos, Nigeria

## Abstract

**Background:**

To study the frequency and pattern of use of complementary and alternative medicine (CAM) in patients with essential hypertension attending a tertiary hypertension clinic.

**Methods:**

Two hundred and twenty-five consecutive hypertensive patients attending the hypertension clinic of the Lagos University Teaching Hospital over a 3-month period were interviewed. Socio-demographic data, duration of hypertension, clinic attendance, current blood pressure, and compliance to conventional medications was documented. CAM utilization was explored using both structured and open-ended questions.

**Results:**

There were 90 (40%) male and 135 (60%) female patients with mean age ± SD overall was 55.1 ± 12.4 years. 88 (39.1%) of the respondents used CAM. Herbal products were the most commonly used CAM type. Amongst the CAM users, the most common herbal product used was garlic (69.3%). Others were native herbs (25%), ginger (23.9%), bitter leaf (*Vernonia amygdalina*) (9.1%), and aloe vera (4.5%). 2.5% used spiritual therapy. There was no difference in the clinical characteristics, socio-economic status, and blood pressure control of CAM users and non-users. Patients who utilized CAM had higher BMI compared with those who did not, but the difference was not statistically significant (mean BMI ± SD of 29.1 ± 5.6 vs 27.1 ± 5.9 kg/m^2^; P = 0.05).

**Conclusion:**

A significant proportion of hypertensive patients attending our tertiary facility and receiving conventional treatment also use CAM therapies. Clinicians need to be aware of this practice, understand the rationale for this health-seeking behaviour, proactively enquire about their use, and counsel patients regarding the potential of some of the therapies for adverse reactions and drug interactions.

## Background

The terms complementary and alternative medicines (CAM) generally refer to practices that are not integral parts of conventional or orthodox medicine, and are consequently not taught as part of the archetypal medical education curriculum [1]. The National Institute of Health (NIH) classifies CAM into five major categories: *alternative medical systems *(e.g. traditional oriental medicine, acupuncture, Ayurveda, naturopathy, homeopathy, Native American healing, Tibetan medicine), *mind-body interventions *(meditation, hypnosis, dance, art and music therapy, spiritual healing, and prayer), *biologic – based therapies *(herbal medicine and dietary supplements, special diets, and orthomolecular medicine), *manipulative and body-based methods *(chiropractic, massage, the Feldenkrais method, other "body work" systems, and aspects of osteopathic medicine such as craniosacral work), *and energy therapies *(reiki, therapeutic touch, and other methods of affecting the "bioelectric field" of the body) [2].

The frequency of utilization of CAM is increasing worldwide, and is well documented in both African and global populations to be between 20 – 80% [1,3-8]. The persuasive appeal of CAM is premised on the fundamental assumptions and principles by which the system operates. These include the presumption that CAM are 'natural', provide the user with a connection to life-supporting forces (vitalism), have a 'scientific basis' and promote 'spirituality' [9,10]. However, considering the lack of evidence to support the efficacy of many of these CAM therapies, the potential for adverse effects, cost considerations, and the trend towards making treatment decisions based on evidence, many medical practitioners entreat caution in the use of CAM.

Specifically, in chronic conditions in which health outcomes are closely linked to adherence to treatment (e.g. hypertension), the use of CAM may potentially adversely affect outcome [11]. Multiple therapy practices involving combined use of CAM (particularly herbal medicines) and prescription medications has also been identified as being prevalent in some populations [12]. The objective of this study was to determine the frequency of utilization of CAM in our hypertensive population, determine if there are any socio-demographic, clinical or treatment-related characteristics that determined CAM use in our patient population, and examine the impact of CAM use on blood pressure control.

## Methods

This descriptive study was carried out in the hypertension clinic of the Lagos University Teaching Hospital, Lagos, Nigeria. The inclusion criteria were diagnosed hypertension and attendance at the hypertension clinic for at least six months. Two hundred and twenty five serially attending, verbally consenting patients with hypertension (HBP) were interviewed using a pre-tested questionnaire. Socio-demographic and hypertension-related data including age, gender, level of educational, monthly income, duration of HBP, duration of current hypertension clinic attendance, BP at initial presentation at the clinic, and current BP (using standard methods were documented [13]. BP control was defined as current BP ≤ 140/90 [13]. Anthropometric indices (weight in kilograms, height in metres, and body mass index in kg/m^2^) were recorded. Obesity was defined as a body mass index ≥ 30 kg/m^2^. Three monthly income groups were used: low income (<N20,000 i.e. <US$150 at N135 = US$1), medium income (≥ N20,000 – 200,000 i.e. ≥ US$150–1500), and high income (≥ N200,000 i.e. ≥ US$1500). Numbers of antihypertensive drugs in individual patients were documented and patients were categorized as being either on monotherapy or combination therapy. Non-compliance to antihypertensive therapy (defined as missing at least 2 days of medication per week) was also documented based on directly questioning the patients [14].

We utilized a standard format to question patients about use of CAM. The questionnaire included a list of commonly used methods, but open-ended questioning was also used to explore the use of other modalities that were not included in the questionnaire. CAM utilization was defined as use of any therapy classifiable as either a complementary or alternative therapy, and categorized on the basis of type of CAM, into the following: healing systems, mind-body connections, dietary supplements and herbs, energy therapy, and manipulation and touch therapy.

### Statistical analysis

Data analysis was conducted using EPIINFO ^® ^2002. Numerical data are expressed as mean values ± standard deviation (SD) and compared using student t test, while categorical variables are expressed as proportions and compared using χ^2 ^test. Statistical significance was assumed at a P value < 0.05.

## Results

The demographic and clinical profiles of the 225 hypertensive patients evaluated are shown in Table 1. The income group distribution was as follows: low income 150 (66.7%), middle income 74 (32.9%), and high income 1 (0.4%). Amongst the patients studied, 39 (17.3%) had no formal education, 60 (26.7%) had primary, 73 (32.4%) secondary, and 53 (23.6%) tertiary education.

**Table 1:** Demographic and clinical characteristics of hypertensives using or not using complementary and alternative therapies

Characteristics	Overall population n = 225 (% or SD)	CAM users n = 88 (% or SD)	CAM non-users n = 137 (% or SD)	Test statistic*	P value
Mean age, years (SD)	55.1 (12.4)	55.2 (10.5)	55.0 (13.5)	0.16	NS
Male/female	90/135	32/56	58/79	0.79	NS
BMI (kg/m^2^)	28.3 (5.5)	29.1 (5.6)	27.1 (5.9)	1.96	0.05
Initial systolic blood pressure mmHg	159.9 (25.4)	158.3 (24.8)	161.0 (25.8)	0.77	NS
Initial diastolic blood pressure mmHg	102.5 (17.4)	100.9 (15.6)	103.5 (18.4)	1.08	NS
Current systolic blood pressure mmHg	141.9 (23.4)	140.4 (22.1)	142.8 (24.3)	0.76	NS
Current diastolic blood pressure mmHg	89.2 (16.2)	87.7 (12.8)	90.1 (18.0)	1.07	NS
% compliant	148 (65.8)	57 (64.8%)	91 (66.4%)	0.06	NS
% Blood pressure controlled	89 (39.6)	35 (39.8%)	54 (39.4%)	0.00	NS
Duration of hypertension (years)	10.8 (8.5)	10.7 (8.7)	10.7 (8.4)	0.02	NS
Duration of clinic attendance (months)	79.5 (77.4)	73.6 (73.1)	83.4 (80.1)	0.78	NS

* Statistical comparison of the CAM users and non-users. Test statistic derived from students T test comparing mean values, and Χ^2 ^test comparing proportions.

CAM complementary and alternative medicine

### Profile of complementary and alternative therapies utilization

CAM was reportedly utilized in 88 (39.1%) hypertensive patients. The specific CAM used (either alone or in combination with other CAM) and their frequencies (amongst the 88 CAM users) were as follows: garlic 61(69.3%), native herbs 22 (25%), ginger 21 (23.9%), bitter leaf (*Vernonia amygdalina*) 8 (9.1%), aloe vera 4 (4.5%) and prayers and fasting 3(3.4%). Based on the classification of CAM, none of the patients used healing systems, energy therapy, or manipulation and touch as CAM for hypertension. The most frequently used CAM category overall was dietary supplements and herbs, documented in 85 (96.6%). Mind-body connections (prayer and fasting only) were used in 3 (3.4%).

Several demographic and clinical parameters were compared in users and non-users of CAM (Table 1). None of the parameters evaluated in this study significantly differed when CAM users were compared to non-users.

## Discussion

It is evident that CAM utilization is on the increase globally, and is being given recognition by health insurance providers in developed countries [1-5]. In our environment, CAM appears to be strongly considered by hypertensive patients as the only viable alternative for a cure for hypertension [15]. In this study we report on the prevalence of CAM usage among hypertensive patients attending a tertiary hypertension clinic in an urban area, and explore some possible patient characteristics determining CAM utilization.

The frequency of CAM use in our study (39.1%) is similar to that reported by other studies. The reported frequencies of CAM use in the general population (irrespective of disease entity) from different parts of the world include 40% in the US [1], 38.5% among the Indian community of Chatsworth in South Africa [7] and 48.5% in Australia [4]. Other studies have reported a higher usage of CAM among their patients [8,12,16]. Specifically amongst hypertensive cohorts, Shafiq et al [16] reported that as many as 63.9% of their hypertensive subjects in a clinic in India took herbal medicines, while in Morocco 80% of patients with hypertension and diabetes used medicinal plants to treat their ailments [8].

In our study, the use of CAM was independent of socio-demographic factors, and was not related to duration of hypertension, duration of clinic attendance or initial BP at presentation. These findings differ from the American and Australian studies, which both found several associations between demographic factors and CAM usage [1,4]. Notably, college education and wealth were predictors common to both the US and Australian studies. Variation in the subject characteristics between our study and these previous studies may account for these differences. The proportion of subjects with tertiary education in our study was lower than in the two previous studies (23.6% compared with 40%) and 66.2% of our subjects were in the low income group category compared with 30% in the other studies [1,4]. Educational level did not have any influence on the use of CAM in our study. Studies have shown that people use CAM to explore the potential benefits purported therein [9] and others use it for chronic illnesses like rheumatologic conditions [17], cancer [18], and depression [3] for which they feel CAM may offer a superior benefit than conventional medicine. In support of this, Oke and Bandele, in a survey on misconceptions about hypertension amongst 1365 Nigerian hypertensives, noted that 21% of the respondents were of the opinion that they would achieve a permanent cure of hypertension only from alternative medicine practitioners, and would consider using alternative medicine in the future [15]. In a survey by Eisenberg and colleagues, the majority of the respondents adjudged that CAM was better than conventional therapy in treatment of rheumatologic conditions [17]. We propose that cultural influences and perceptions about the efficacy of CAM may be more important determinants of its use (as observed by Clement et al in Trinidad) and may override socioeconomic factors such as educational level and economics in our environment [19].

Several reasons have been given by researchers for the increased prevalence of CAM utilization. These include failure of modern medicine to cure the underlying problem [9], and the perception that CAM is cheaper than conventional medicines [8]. Some other attractions to alternative therapies may be related to the power of the underlying philosophies they share, which involve closeness to nature, spirituality, and the fact that these therapies often go along with the cultural beliefs of the people [9,10].

Although we did not enquire from users why they took CAM, other possible reasons for use of CAM in our environment may include the strong advertisements by alternative practitioners that CAM is a panacea to all diseases thus encouraging patients to try them out. Added to this are side effects of some hypertensive medications (especially on erectile function in men). Some patients may therefore resort to these natural forms of therapy, presumably with no side effects. Side effects were cited as one of the reasons for the use of CAM by 59% of the respondents in the study by Shafiq et al [16]. Another possible explanation is the cultural beliefs of Africans that illnesses have a 'spiritual' origin. Patients are thus interested in finding an explanation for their symptoms or the root cause of their problems and therefore consult with alternative practitioners. In Nigeria the use of herbal remedies, perceived to be cheaper, may be on the increase due to the poor economic state and the increasing costs of orthodox medicines. For instance, garlic, widely used for treating hypercholesterolemia (despite conflicting scientific data) is far less expensive than the statins [20,21]. Furthermore, many of the most commonly used supplements like garlic, ginger and aloe vera are also used routinely as food spices and beverages, thus increasing their acceptability by consumers [2,20]. Of note is the higher BMI in those who took CAM. The precise reason for this was not explored in our study. However, this may be based on adverts that state that some herbal products aid weight reduction.

The most commonly used CAM type in our study were herbs. This is similar to reports by Singh et al [7] but differs from reports by Shafiq et al [16] in which ayurveda therapy was the most common type of CAM used. Before the advent of orthodox medicine in Nigeria, herbal medicines had been the mainstay of treatment for various ailments and were dispensed by traditional herbalists involved in their cultivation and preparation. The observed high use of herbal remedies in our study may be linked to this cultural background and history. Garlic was the most frequently used herbal CAM. Historically, garlic has been shown to reduce cardiovascular events by lowering plasma cholesterol, blood pressure and by inhibiting platelet aggregation [20,21]. Allicin is the ingredient responsible for the lipid-lowering and anti-platelet effect of garlic [21]. However, there are conflicting results about the efficacy of garlic in improving cardiovascular function in the literature [20,21]. This is due to the different forms of garlic used as this affects the amount of the active ingredient present. Secondly, some the other forms of CAM therapies (such as acupuncture and chiropractic) are less well known in our environment, and where available, may not be affordable to the predominantly low income patients in this study.

In the present study, the compliance rate to conventional anti-hypertensive drugs was similar among patients who used CAM and those who did not, thus indicating that most patients used alternative therapies along with their orthodox medications. Studies have shown that majority of CAM users use it along with their orthodox medications [7,8,12,16]. Likewise, the BP control did not differ among the two groups of patients. This may indicate that the CAM used by our patients did not significantly affect their BP control, or conversely, that the magnitude of CAM use was insufficient to have any deleterious effect on BP control.

Despite the fact that herbal supplement use is increasing, there is a lack of rigorous evidence to support their efficacy. Some supplements (e.g. ephedra) have serious, sometimes devastating side effects [2]. Thus, healthcare providers should be familiar with the most common herbal medicines, especially their potential for adverse effects and drug interactions and therefore can advise patients against their use. The problems with alternative medicines are lack of standardization of doses, possible drug interactions with conventional medicines and side effects. Some herbs, including garlic, ginkgo, ginseng, and St John's wort, can have a significant influence on concurrently administered drugs [2,20,22]. Herbal medicines may mimic, decrease, or increase the action of prescribed drugs [22]. This can be especially important for drugs with narrow therapeutic windows and in sensitive patient populations such as older adults, the chronically ill, and those with compromised immune systems [2]. Many herbal remedies have not undergone careful scientific assessment, and some have the potential to cause serious toxic effects and major drug-to-drug interactions. With the high prevalence of herbal use among our hypertensive patients it is important that attending clinicians must inquire about such health practices for hypertension, diabetes mellitus, cardiac disease and liver diseases and be informed about the potential for benefit and harm. Continuing research is necessary to elucidate the pharmacological activities of the many herbal remedies now being used to treat cardiovascular diseases.

## Conclusion

The use of CAM is quite common among hypertensive patients attending the hypertension clinic of our hospital and usage is independent of socio-demographic factors. Cultural and economic reasons are largely responsible for the use of CAM. While not totally harmful, doctors must equip themselves with knowledge of the common CAM therapies and also remember to ask their patients whether or not they use CAM.

## Competing interests

The author(s) declare that they have no competing interests.

## Authors' contributions

COA conceived of the study and participated in its design, coordination, data collection, data analysis, drafting of the manuscript and approval of the final manuscript. NUO participated in the design, coordination, data collection, statistical analysis, drafting of the manuscript and approval of the final manuscript.

## Pre-publication history

The pre-publication history for this paper can be accessed here:


